# A unique DNA-binding mode of African swine fever virus AP endonuclease

**DOI:** 10.1038/s41421-020-0146-2

**Published:** 2020-03-17

**Authors:** Yiqing Chen, Xi Chen, Qi Huang, Zhiwei Shao, Yanqing Gao, Yangyang Li, Chun Yang, Hehua Liu, Jixi Li, Qiyao Wang, Jinbiao Ma, Yong-Zhen Zhang, Yijun Gu, Jianhua Gan

**Affiliations:** 1 State Key Laboratory of Genetic Engineering, Collaborative Innovation Center of Genetics and Development, Shanghai Public Health Clinical Center, School of Life Sciences, Fudan University, 200438 Shanghai, China; 2 State Key Laboratory of Bioreactor Engineering, East China University of Science and Technology, 200237 Shanghai, China; 3 Shanghai Public Health Clinical Center, School of Life Sciences, Fudan University, 200438 Shanghai, China; 4 State Key Laboratory for Infectious Disease Prevention and Control, Collaborative Innovation Center for Diagnosis and Treatment of Infectious Diseases, National Institute for Communicable Disease Control and Prevention, Chinese Center for Disease Control and Prevention, Changping, 102206 Beijing, China; 5 National Center for Protein Science Shanghai, Shanghai Advanced Research Institute, Chinese Academy of Sciences, 201210 Shanghai, China

**Keywords:** X-ray crystallography, Base excision repair

## Abstract

African swine fever virus (ASFV) is highly contagious and can cause lethal disease in pigs. ASFV is primarily replicated in the cytoplasm of pig macrophages, which is oxidative and caused constant damage to ASFV genome. ASFV AP endonuclease (*Asfv*AP) catalyzes DNA cleavage reaction at the abasic site and is a key enzyme of ASFV base excision repair (BER) system. Although it plays an essential role in ASFV survival in host cells, the basis underlying substrate binding and cleavage by *Asfv*AP remains unclear. Here, we reported the structural and functional studies of *Asfv*AP, showing that *Asfv*AP adopts a novel DNA-binding mode distinct from other APs. *Asfv*AP possesses many unique structural features, including one narrower nucleotide-binding pocket at the active site, the C16–C20 disulfide bond-containing region, and histidine-rich loop. As indicated by our mutagenesis, in vitro binding and cleavage assays, these features are important for *Asfv*AP to suit the acidic and oxidative environment. Owing to their functional importance, these unique features could serve as targets for designing small molecule inhibitors that could disrupt the repair process of ASFV genome and help fight against this deadly virus in the future.

## Introduction

African swine fever virus (ASFV) is a large double-stranded DNA (dsDNA) virus, and also the only member of the *Asfarviridae* family. ASFV is highly contagious^1^ and can cause lethal disease in pigs^2^. To date, ASFV has been found in many countries of the world and has caused a substantial loss in agricultural industry since its first report in Kenya in 1921^3^. To prevent a nationwide epidemic, more than 500,000 pigs were killed in Cuba in 1971, which was labeled the “most alarming event” of 1971 by the United Nations Food and Agricultural Organization^4^. In 2011, the virus killed over 300,000 pigs in the Russian Federation region. Since the first report in August 2018^5^, ASFV has been reported in over 30 provinces in China, caused huge economic losses, as well as immediate pork shortage. Due to its serious threat to the agricultural industry, ASFV has attracted tremendous attention from governments and scientists in the past decade in the world. Unfortunately, no vaccine or other useful treatment against this virus has been developed so far^6^.

ASFV is one of the most complex viruses known to date. Its genome varies between 170 and 190 kb, encoding more than 150 proteins that are involved in various stages of ASFV life cycle, including suppression of host immune response, entry into host cells, gene expression, and virion assembly^7^. ASFV is primarily replicated in the cytoplasm of swine macrophage cells^8^. The cytoplasm of macrophages is very rich in free oxygen radicals that caused constant damages to ASFV genome^9,10^. To efficiently overcome these damages, especially for DNA abasic sites (AP sites), ASFV evolved its own base excision repair (BER) system, including an AP endonuclease (*Asfv*AP), a repair polymerase (*Asfv*PolX), and a ligase (*Asfv*LIG). Unlike other repair enzymes, the fidelities of both *Asfv*PolX^11^ and *Asfv*LIG^12^ are very low. Therefore, in addition to maintaining the integrity of viral genome, these enzymes may also play important roles in strategic mutagenesis^11^ and genotype formation of ASFV, which further complicated the epidemiology, diagnosis, and prevention of ASFV^13–15^.

Owing to their functional importance, ASFV repair enzymes have been extensively studied^16,17^. The NMR structures of *Asfv*PolX were independently reported by Tsai and Mullen previously^18–20^. The crystal structures of both *Asfv*PolX^21^ and *Asfv*LIG^22^ were determined by our group very recently. In addition to the intrinsic mechanism of DNA repair, these structures also unraveled many features unique to ASFV repair enzymes, such as the 5′ phosphate group (5′-P) binding pocket located at the finger domain of *Asfv*PolX and the DNA-binding domain located at the N-terminus of *Asfv*LIG.

**Fig. 1:**
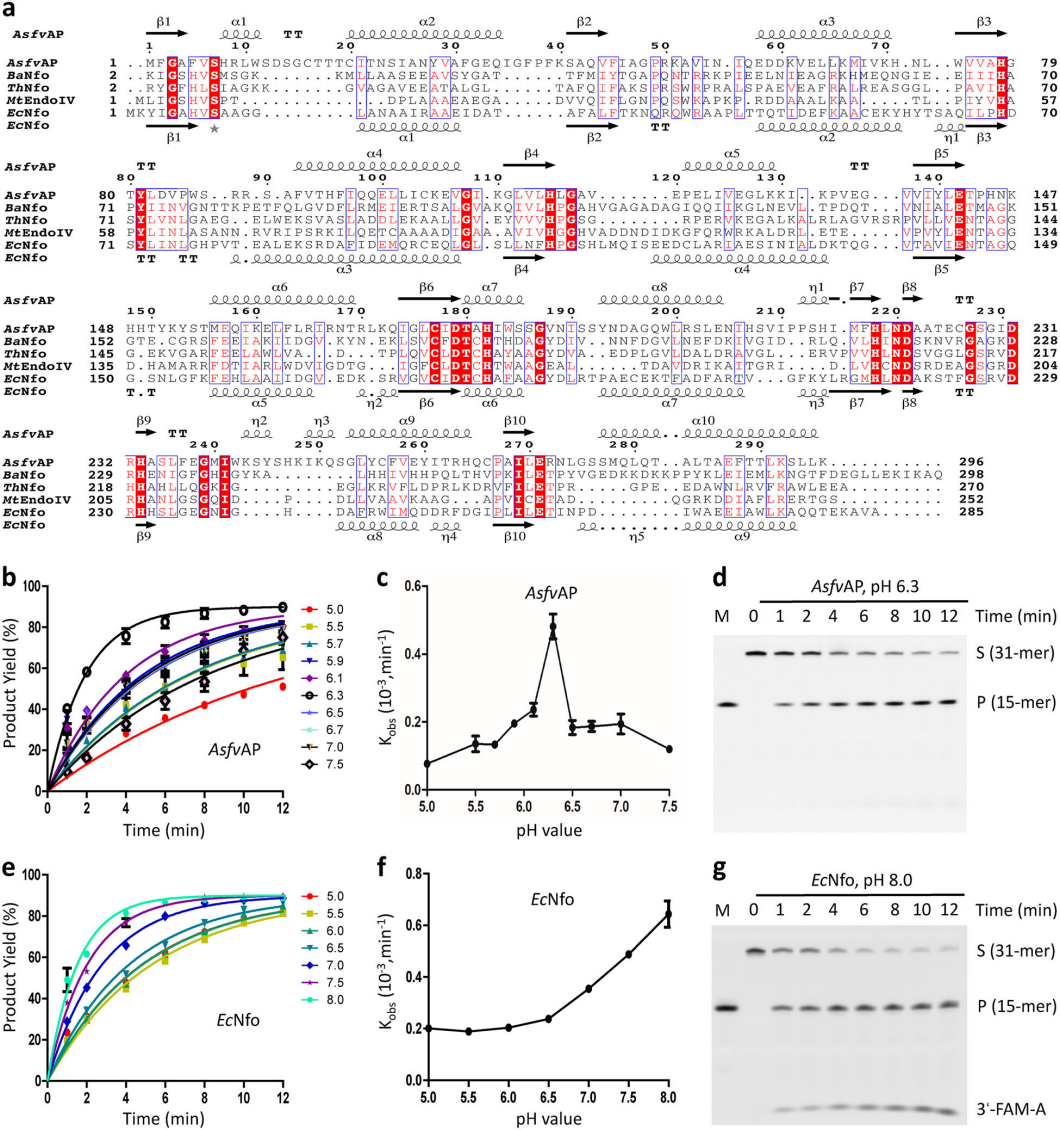
Comparison of AP endonuclease activities of *Asfv*AP and *Ec*Nfo. **a** Sequence alignment of *Asfv*AP and homologous proteins. **b** Impacts of pH on the AP endonuclease activity of *Asfv*AP. **c** Comparison of the *K*_obs_ values of *Asfv*AP under different pH. **d** SDS-PAGE gel analysis showing DNA cleavage by *Asfv*AP under pH 6.3. **e** Impacts of pH on the AP endonuclease activity of *Ec*Nfo. **f** Comparison of the *K*_obs_ values of *Ec*Nfo under different pH. **g** SDS-PAGE gel analysis showing DNA cleavage by *Ec*Nfo under pH 8.0. FAM-labeled DNA-3 (5′-GGTAAGGGCAGCGTCCFCGACGAGGAATGCA-FAM-3′, 3′-CCATTCCCGTCGCAGGGGCTGCTCCTTACGT-5′) was used in all AP endonuclease assays. The substrate and product bands are labeled by S and P, respectively. DNA marker (5′-FCGACGAGGAATGCA-FAM-3′) is labeled as M. In panels **b**, **c** and **e**, **f**, the data represent the mean of three independent experiments. The standard deviation (±SD) values are indicated by error bars. All SDS-Page gel analysis were repeated for at least three times, the representative gels were shown in panels **d, g**.

*Asfv*AP belongs to the class II AP endonuclease family; it mainly catalyzes DNA cleavage reaction at the 5′ side of abasic site (AP), generating 3′-OH group and 5′-deoxyribose phosphate (dRP)^23^. *Asfv*AP-catalyzed reaction represents one key step in ASFV BER pathway. Deletion of *Asfv*AP-coding gene (pE296R) lowered the growth rate of the virus to 2–4% of the parental virus in swine macrophages^17^. *Asfv*AP shares very low sequence identity (~10%) with the homologous proteins including *E. Coli* Nfo (*Ec*Nfo), *G. Kaustophilus* Nfo (*Gk*Nfo), *B. Anthracis* Nfo (*Ba*Nfo), *T. Thermus* Nfo (*Tt*Nfo), and *M. tuberculosis* EndoIV (*Mt*EndoIV) (Fig. 1a). Similar to *Ec*Nfo, *Asfv*AP possesses 3′→5′ exonuclease, 3′-phosphodiesterase, 3′-phosphase and weak nucleotide incision repair (NIR) activities. However, compared with *Ec*Nfo and other homologous proteins, *Asfv*AP is more specific for the abasic sites^24,25^. DNA binding and cleavage activities of *Asfv*AP are very sensitive to the redox environment^25^. Due to the lack of structural data, the overall folding, DNA recognition, and the basis for redox-sensing of *Asfv*AP remain unclear. Herein we report two complex structures of *Asfv*AP (*Asfv*AP/DNA-1 and *Asfv*AP/DNA-2, Supplementary Table S1), showing that *Asfv*AP adopts one novel DNA-binding mode. Via mutagenesis and in vitro binding and cleavage assays, we also explored the functional roles of the unique features of *Asfv*AP. Our studies not only advance our understanding on ASFV BER pathway but also provide potential basis for anti-ASFV drug designing.

## Results

### pH-dependence of AP endonuclease activity

Normally, macrophage cells acidify their cytoplasm environment to clear intruders^26^. To test the potential effects of acidification on the catalytic efficiency of *Asfv*AP, we designed one FAM-labeled DNA (DNA-3: 5′-GGTAAGGGCAGCGTCCFCGACGAGGAATGCA-FAM-3′, 3′-CCATTCCCGTCGCAGGGGCTGCTCCTTACGT-5′, the abasic site was shown as capital letter F in the sequence) and performed in vitro cleavage assays. As depicted in Fig. 1b–d and Supplementary Fig. S1, wild type (WT) *Asfv*AP could cleave DNA-3 within a wide range of pH. The highest activity was observed under pH 6.3, and the apparent rate constant (*K*_obs_) was about 0.55 × 10^−3^ min^−1^. Increasing the buffer pH to 7.5 or decreasing it to 5.0 caused 5–7-fold reduction in the catalytic efficiency of *Asfv*AP, but the protein retained 40% activity when the pH varied between 5.9 and 7.0 (Supplementary Table S2).

The above observations suggested that *Asfv*AP prefers weak acidic condition for its AP endonuclease activity. The sequence identities between *Asfv*AP and the homologous proteins are very low (Fig. 1a). For direct comparison, we purified *Ec*Nfo and performed in vitro cleavage assays. As depicted in Fig. 1e–g and Supplementary Fig. S2, *Ec*Nfo could cleave DNA-3 within a wide range of pH. The K_obs_ value of *Ec*Nfo was around 0.20 × 10^−3^ min^−1^ under pH 5.0–6.5, and increased to 0.49 × 10^−3^ min^−1^ and 0.56 × 10^−3^ min^−1^ at pH 7.5 and 8.0 (Supplementary Table S3), respectively. Different from *Asfv*AP, the cleavage assay results suggested that *Ec*Nfo prefers basic condition for its AP endonuclease activity. In addition to the main abasic site cleavage products, some minor products were also produced by *Ec*Nfo under pH 7.0–8.0 (Fig. 1g and Supplementary Fig. S2). These minor products should correspond to 3′-FAM-A, which were generated by the 3′→5′ exonuclease activity of *Ec*Nfo. Unlike *Ec*Nfo, *Asfv*AP did not show obvious 3′→5′ exonuclease activity under all the tested conditions (Supplementary Fig. S1). Taken together, these data suggested that *Asfv*AP has higher abasic site selectivity.

### Overall folding of the *Asfv*AP/DNA-1 complex

**Fig. 2:**
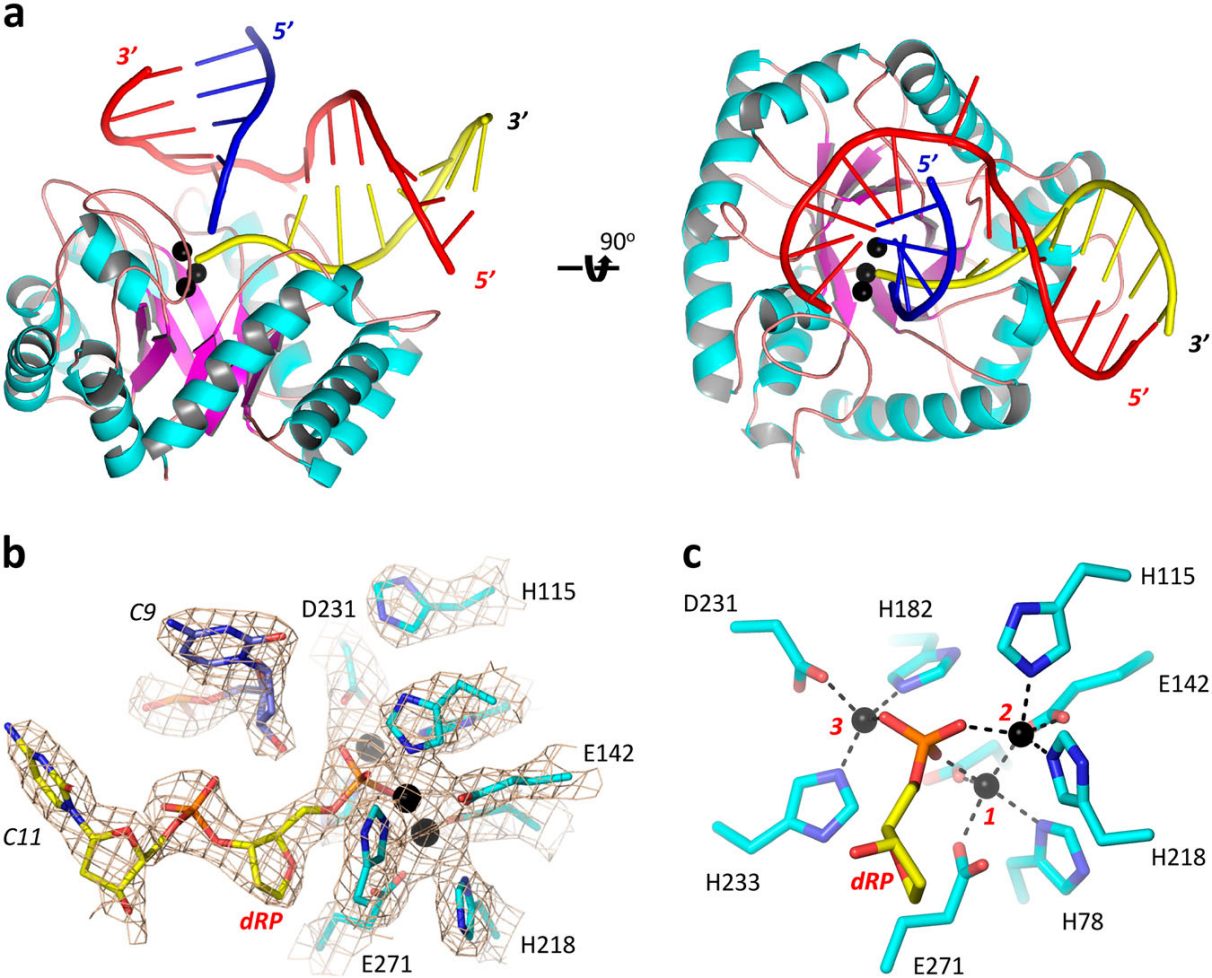
Structure of *Asfv*AP/DNA-1 complex. **a** Cartoon view showing the overall fold of *Asfv*AP/DNA-1 complex. α-helices, β-strands and loops of *Asfv*AP are colored in cyan, purple, and pink, respectively. The uncleaved strand, the upstream and downstream of the product strand of DNA-1 are colored in red, blue and yellow, respectively. **b** Conformations of the active site residues and nucleotides. 2F_o_–F_c_ electron density map was contoured at 1.5 sigma level. **c** Detailed coordination of the Zn^2+^ ions. The Zn^2+^ ions are shown as spheres in black in all panels.

To unravel the molecular basis underlying substrate binding and cleavage by *Asfv*AP, we performed crystallographic studies and solved two complex structures of *Asfv*AP, *Asfv*AP/DNA-1, and *Asfv*AP/DNA-2. Both DNA-1 (5′-GCAGCGTCCFCGACGAGG-3′ and 3′-CGTCGCAGGGGCTGCTCC-5′) and DNA-2 (5′-GCAGCGTCACCGACGAAA-3′ and 3′-CGTCGCAGGGGCTGCTCC-5′) are 18-bp dsDNAs, containing one abasic site and one A:G mispair in the middle, respectively. *Asfv*AP/DNA-1 crystal belongs to P2_1_ space group. Per asymmetric unit contains two *Asfv*AP/DNA-1 complexes, and each complex is formed by one *Asfv*AP protein and one DNA-1 molecule (Fig. 2a). *Asfv*AP is of α/β fold in nature; the β-strands form one β-barrel in the center, surrounded by α-helices at the outer side. DNA molecule adopts regular B-form-like conformations at both ends, but it was severely bent with the angle between the two helical axes being about 100° in the middle.

Intact DNA-1 duplex was utilized in the crystallization process. However, as confirmed by the clear 2F_o_–F_c_ electron density maps, the DNA was cleaved at the 5′ side of the abasic site in the structure (Fig. 2b). Three well-defined Zn^2+^ ions were captured in the structure, coordinating with the side chains of His78, His115, Glu142, Asp179, His182, Asp231, His233, and Glu271. In addition, the Zn^2+^ ions also forms direct coordination with the 5′-phosphate group of dRP, the product of the abasic site cleavage (Fig. 2c). Taken together, these observations demonstrated the catalytic activity of *Asfv*AP and indicated that the *Asfv*AP/DNA-1 complex was assembled in a catalytic form.

### Molecular basis for target DNA recognition by *Asfv*AP

**Fig. 3:**
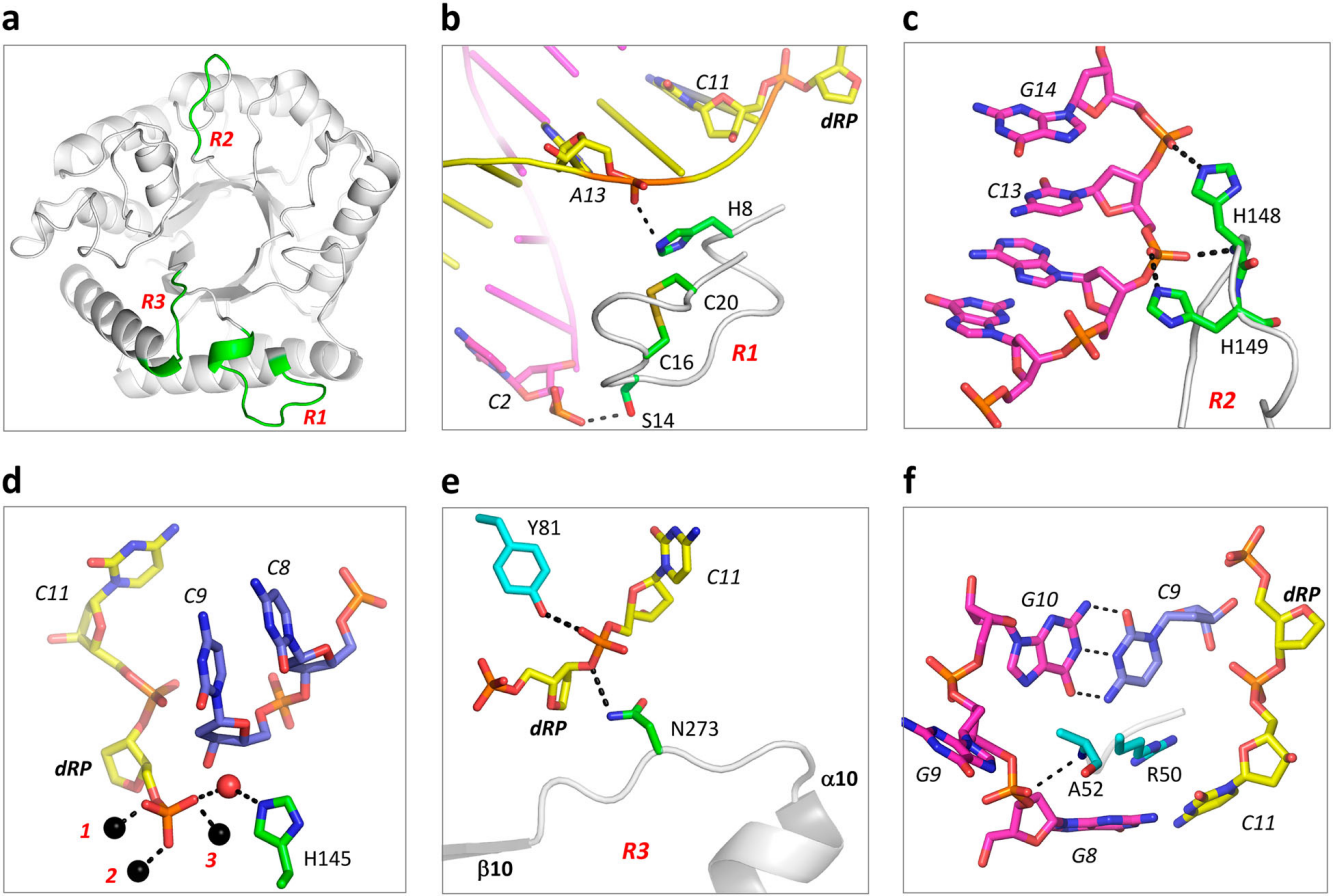
Protein-DNA interactions observed in the *Asfv*AP/DNA-1 complex. **a** Cartoon view showing the conformations and relative orientations of the R1, R2 and R3 regions, which are colored in green. **b** Detailed interactions between DNA-1 and the R1 region. DNA-1 and R1 are shown as cartoon. The interacting residues (His8 and Ser14) and nucleotides (A13 and C2) are highlighted by sticks. dRP nucleotide of DNA-1 and Cys16-Cys20 disulfide bond of R1 are also highlighted by sticks. **c**–**d** Detailed interactions between DNA-1 and the R2 region. Zn^2+^ ions and water molecule are shown as spheres in black and red, respectively. **e** Detailed interactions between DNA-1 and the R3 region. Besides Asn273 of the R3 region, the conserved Tyr81 residue is also highlighted by sticks. **f** Interactions between nick region nucleotides and *Asfv*AP residues.

The *Asfv*AP/DNA-1 complex structure was refined at atomic resolution (2.35 Å), which revealed the detailed basis for target DNA recognition by *Asfv*AP. The DNA molecule mainly interacts with *Asfv*AP in three regions: R1, R2, and R3 (Fig. 3a). The R1 region (_8_HRLWSDSGCTTTC_20_) is composed of one short α-turn immediately followed by a loop. The R2 region is one histidine-rich loop (_145_HNKHHT_150_), connecting β5 strand and α6 helix at the central region. The R3 region (_272_RNLGSSMQLQ_281_) consists of the β10-α10 linker and the N-terminal half of α10 helix at the C-terminus. R1 and R3 are close to each other, whereas R2 is isolated in space.

The conformation of the target DNA was stabilized by several different types of interactions. The R1 region (Fig. 3b) mainly interacts with DNA through hydrogen binding (H-bond). The NE2 atom of His8 (the first residue of R1) forms one H-bond with the phosphate group of A13 of the product strand; A13 is 3-nt downstream of dRP. Ser14 is located at the middle of R1, and also forms one direct H-bond with DNA via its side chain. However, instead of the product strand, it interacts with C2 near the 5′-end of the uncleaved strand.

Of the six residues of the R2 loop, three are histidines, including His145, His148, and His149. Both His148 and His149 locate at the tip region of the loop and directly interact with the uncleaved DNA strand (Fig. 3c). Via its main chain N atom and the side chain NE2 atom, His148 forms two H-bonds: one with the OP1 atom of C13 and another with the OP2 atom of G14. Obviously, the NE2 atom of His149 forms one H-bond with the OP2 atom of C13. His145 resides at the N-terminus of the R2 loop. Although it does not form direct H-bond interaction with the DNA, it interacts with the 5′-phosphate group of dRP through one water molecule (Fig. 3d).

The side chain of Asn273 of R3 points toward the product strand (Fig. 3e). The ND2 atom of Asn273 forms one H-bond with the O3′ atom of dRP, stabilizing the dRP conformation from the 3′ side. The conformation of C11, located at the right downstream of dRP, was stabilized by two types of interactions: the OP2 atom of C11 interacts with hydroxyl group of the side chain of Tyr81 (Fig. 3e) and the nucleobase of C11 stacks against the guanidine group of Arg50 (Fig. 3f). The side chain of Arg50 is deeply inserted into the minor grove of the duplex, which is most likely the major factor for the significant bending of the DNA. The phosphate group of G9 of the uncleaved strand forms one H-bond with the main chain N atom of Ala52. However, due to lacking of pairing with other nucleotide, the nucleobase of G9 was flipped out.

### A novel DNA-binding mode of *Asfv*AP

**Fig. 4:**
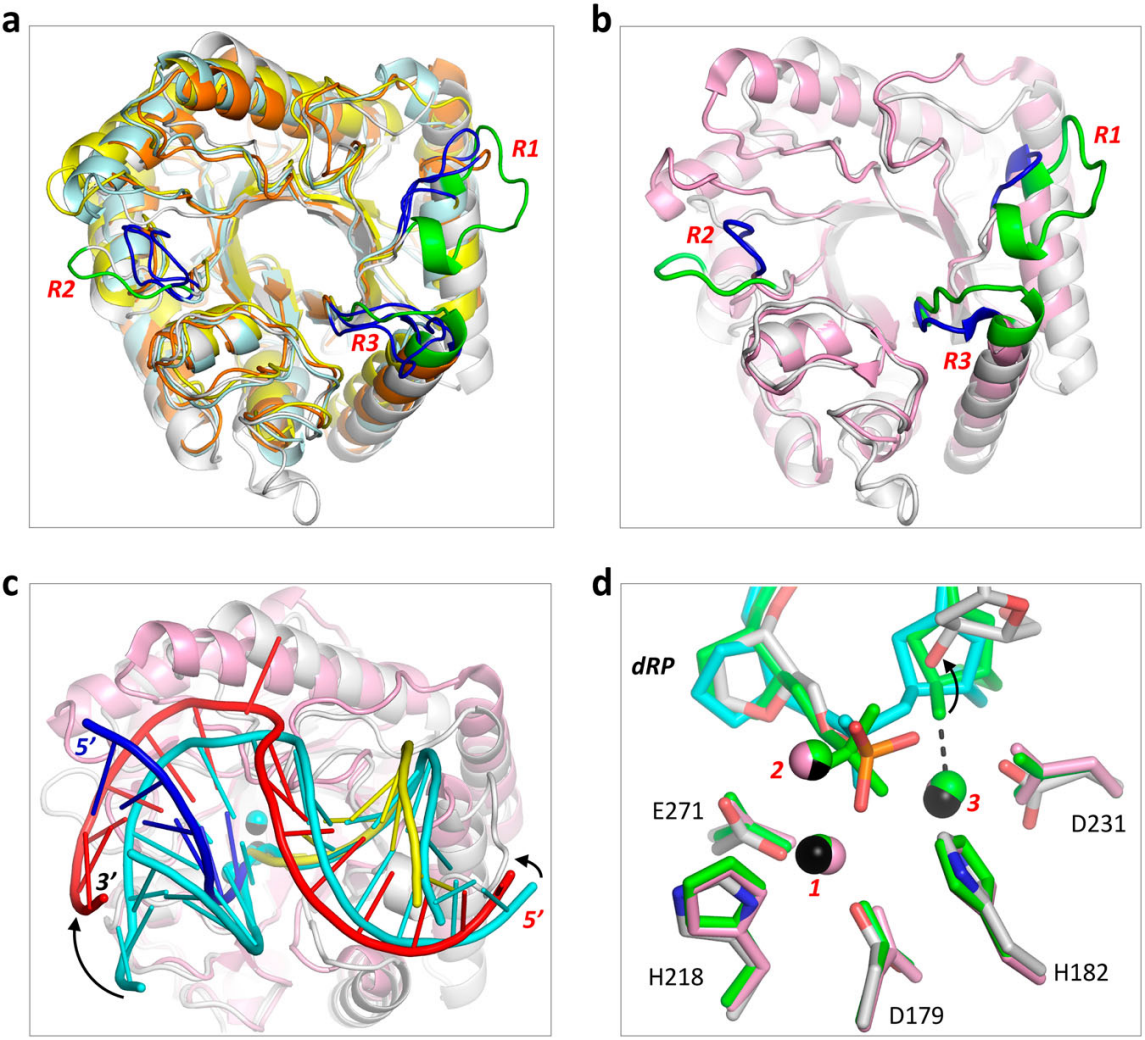
Structural comparison between *Asfv*AP and homologous proteins. **a** Superposition of *Asfv*AP, *Ba*Nfo, *Mt*EndoIV, and *Tt*Nfo structures. **b**, **c** Superposition of *Asfv*AP/DNA-1 and *Ec*Nfo/substrate (PDB: 2NQJ) complexes. **d** Superposition of the DNAs bound in the active sites of *Asfv*AP/DNA-1, *Ec*Nfo/product (PDB: 2NQ9), and *Ec*Nfo/substrate complexes. In panels **a**, **b**, *Asfv*AP, *Ba*Nfo, *Mt*EndoIV, *Tt*Nfo, and *Ec*Nfo are colored in white, cyan, yellow, orange, and pink, respectively. The R1–R3 regions are colored in green for *Asfv*AP, whereas they are colored in blue for all other proteins. In panel **c**, *Asfv*AP and *Ec*Nfo are colored in white and pink, respectively. For the *Asfv*AP/DNA-1 complex, the uncleaved strand, upstream and downstream of the cleaved strand are colored in red, blue and yellow, respectively. Zn^2+^ ions are shown as black spheres. For the *Ec*Nfo/substrate complex, DNA strands and Zn^2+^ ions are all colored in cyan. In panel **d**, DNA, protein, and Zn^2+^ ions are colored in atomic colors (C, white; N, blue; O, red; P, orange; Zn^2+^, black) for the *Asfv*AP/DNA-1 complex. The *Ec*Nfo/product complex is colored in green. For the *Ec*Nfo/substrate complex, DNA is colored in cyan, protein and Zn^2+^ ions are colored in pink.

Comparison with the reported class II AP endonuclease structures showed that *Asfv*AP has similar secondary structure topology as *Ba*Nfo, *Mt*EndoIV^27^, and *Tt*Nfo^28^, in which the central β-barrels can superimpose with each other very well (Fig. 4a and Supplementary Fig. S3). However, the orientations of the outer α-helices of *Asfv*AP are significantly different from those of *Ba*Nfo, *Mt*EndoIV, and *Tt*Nfo; the root mean square deviation (rmsd) values between *Asfv*AP and the three homologous structures are all greater than 4.0 Å. In addition to apo-structure, several complex structures of *Ec*Nfo have also been reported previously^29,30^. Compared to *Asfv*AP, the overall conformation of *Ec*Nfo is more similar to those of *Ba*Nfo, *Mt*EndoIV, and *Tt*Nfo, might due to the higher sequence identities between them (Fig. 1a). The rmsd value between substrate-bound *Ec*Nfo (PDB: 2NQJ) and *Tt*Nfo is 1.7 Å and only 1.3 Å between *Ec*Nfo and *Ba*Nfo, suggesting that these proteins may share similar mode in DNA binding. *Asfv*AP mainly binds DNA at the R1, R2, and R3 regions (Fig. 3). However, as depicted in Fig. 4a, b, the conformations of R1, R2, and R3 in *Asfv*AP are significantly different from those of the homologous proteins.

Besides the conformational difference between the proteins, structural superposition also revealed significant conformational differences of the DNAs (Fig. 4c). In the *Ec*Nfo/substrate complex structure, DNA forms several stable H-bond interactions with *Ec*Nfo. However, instead of the side chains, these interactions are mainly mediated by the main chains of *Ec*Nfo. Compared with the DNA in the *Ec*Nfo/substrate complex, the DNA in *Asfv*AP/DNA-1 structure is rotated about 10° anti-clockwise and 30° clockwise toward the 5′ and 3′ ends of the uncleaved strand, respectively. The cleavage site dRPs adopt similar conformations in *Asfv*AP/DNA-1, *Ec*Nfo/product (PDB: 2NQ9), and *Ec*Nfo/substrate complexes (Fig. 4d). Like the *Ec*Nfo/substrate complex, the 3′-hydroxyl group did not leave the active site and coordinated with the catalytic Zn^2+^ ion in the *Ec*Nfo/product complex, representing a product-bound state immediately after the cleavage reaction. Compared to the product and substrate complexes of *Ec*Nfo, the 3′-hydroxyl group was shifted away by ~1.7 Å in the *Asfv*AP/DNA-1 complex, which may represent one product-releasing state. The conformations of the catalytic Zn^2+^ ions and the coordinating residues are almost identical in the three complex structures (Fig. 4d). Taken together, these observations suggested that *Asfv*AP adopts a novel DNA-binding mode, but shares the same catalytic mechanism with other class II AP endonucleases.

### R1 and R2 regions are important for *Asfv*AP function

**Fig. 5:**
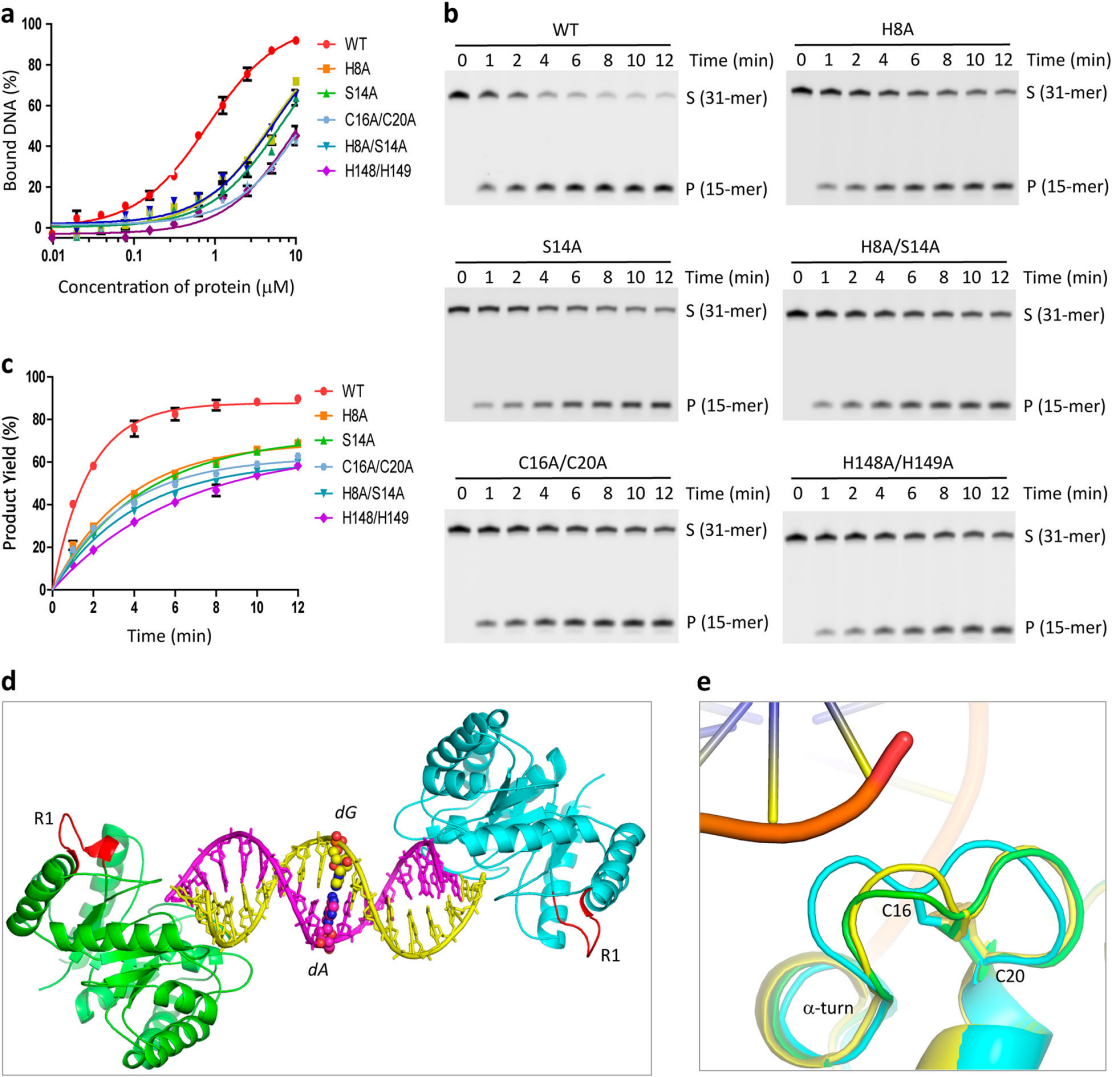
Verification of the functional importance of R1 and R2 regions. **a** Comparison of DNA binding by WT and mutated *Asfv*AP. **b, c** SDS-PAGE gel analysis and comparison of DNA-3 cleavage by WT and mutated *Asfv*AP. The substrate and product bands are labeled by S and P, respectively. **d** Overall fold of the *Asfv*AP/DNA-2 complex. The two *Asfv*AP molecules are colored in green and cyan, respectively. The R1 regions are colored in red. dA:dG mispair is highlighted by spheres. **e** Superposition of the R1 regions observed in the *Asfv*AP/DNA-1 and *Asfv*AP/DNA-2 complexes. For the *Asfv*AP/DNA-1 complex, DNA and the R1 region are colored in orange and yellow, respectively. For the *Asfv*AP/DNA-2 complex, the R1 regions are colored cyan and green. The Cys16-Cys20 disulfide bonds are highlighted by sticks in both complexes. In panels **a** and **c**, the data represent the mean of three independent experiments. The standard deviation (±SD) values are indicated by error bars. All SDS-Page gel analysis were repeated for at least three times, the representative gels were shown in panel **b**.

As described above, *Asfv*AP adopts a novel DNA-binding mode and the majorities of the DNA-interacting residues are unique to *Asfv*AP (Figs. 1a and 3). To verify the functional importance of these residues, we constructed several *Asfv*AP mutants and performed in vitro DNA binding and cleavage assays (Fig. 5a–c and Supplementary Table S4). WT *Asfv*AP has strong DNA binding affinity, indicated by the low dissociation value (*K*_d_, 0.81 ± 0.14 μM). Compared to WT *Asfv*AP, substitution of either His8 or Ser14 with Ala lowered the DNA binding affinity of the protein by 6~8-folds. H8A/S14A double mutation led to more than 12-fold reduction in DNA binding. In consistent with their weaker DNA binding affinities, the DNA-3 cleavage activities of H8A, S14A, and H8A/S14A mutants are all weaker than that of WT *Asfv*AP by 2-folds.

Compared to the homologous proteins, the R1 region (aa 8–20) of *Asfv*AP is significantly longer. In *Ec*Nfo, the R1 region is substituted by one 4-amino-acid (_10_AAGG_13_) loop, which adopts one different conformation (Fig. 4b). In addition to single-point mutants, we also constructed one *Asfv*AP mutant (*Asfv*AP_Ec-loop) with the R1 region replaced by AAGG. As depicted in Supplementary Fig. S4, the loop replacement only caused 2-fold reduction in the protein’s DNA binding affinity. However, it weakened the DNA cleavage activity of the protein by 12-folds (Supplementary Table S4). This observation indicated that the R1 region of *Asfv*AP could not be replaced by the corresponding loop of *Ec*Nfo.

*Asfv*AP contains 7 cysteine residues, including Cys16, Cys20, Cys104, Cys177, Cys226, Cys256, and Cys266. Previous studies suggested that *Asfv*AP forms one intramolecular S–S bond^25^. Reducing reagents, such as dithiothreitol (DTT) and β-mercaptoethanol (β-ME), can inhibit DNA binding and cleavage by *Asfv*AP. The inhibitory effects of DTT and β-ME can be reversed by H_2_O_2_, which is oxidative. As revealed by our *Asfv*AP/DNA-1 complex, the S-S bond was formed between Cys16 and Cys20 within the R1 region. Surprisingly, although the S–S bond is important for *Asfv*AP function, neither Cys16 nor Cys20 interacted with the DNA in the structure (Fig. 3). Puzzled by these observations, we performed thoroughly structural comparison and mutagenesis studies. In the *Asfv*AP/DNA-2 structure, each DNA was bound by two *Asfv*AP molecules, but the DNA did not interact with any R1 residues (Fig. 5d). Cys16 could undergo subtle conformational changes, whereas the conformation of the neighboring α-turn and Cys20 of R1 were well fixed in *Asfv*AP/DNA-1 and *Asfv*AP/DNA-2 complexes (Fig. 5e). The DNA binding and cleavage activities of C16A/C20A double mutant are approximately 6-fold and 2-fold weaker than those of WT *Asfv*AP (Fig. 5a–c and Supplementary Table S4). The cytoplasm of macrophages is very rich in free oxygen radicals, which play a similar role as H_2_O_2_ in favoring S-S bond formation. We believed that the S-S bond of *Asfv*AP is evolved to help the virus to adapt the oxidative condition of macrophages.

The lengths of R2 are comparable in *Asfv*AP and the homologous proteins. However, the sequence of *Asfv*AP R2 is very different from those of the homologous proteins (Fig. 1a). In *Asfv*AP/DNA-1 complex, the R2 residues His148 and His149 form direct H-bond interactions with DNA-1 (Fig. 3c). To investigate the potential roles of the histidine residues, one H148A/H149A double mutant was constructed and its DNA binding affinity and enzymatic activity were measured. Compared to WT *Asfv*AP, the DNA binding and cleavage activities of H148A/H149A mutant are approximately 15-fold and 3.5-fold weaker (Fig. 5a–c and Supplementary Table S4). Like His148 and His149, His8, and His145 are also involved in DNA binding (Fig. 3). It is well known that histidine is quite different from other residues, and that its protonation state is very sensitive to the pH value of the environment. We speculated that these histidine residues are evolved to enhance the catalytic efficiency of *Asfv*AP (Fig. 1b, c) and to help the virus survive under the acidic condition of macrophages.

### One narrower nucleotide-binding pocket in *Asfv*AP

**Fig. 6:**
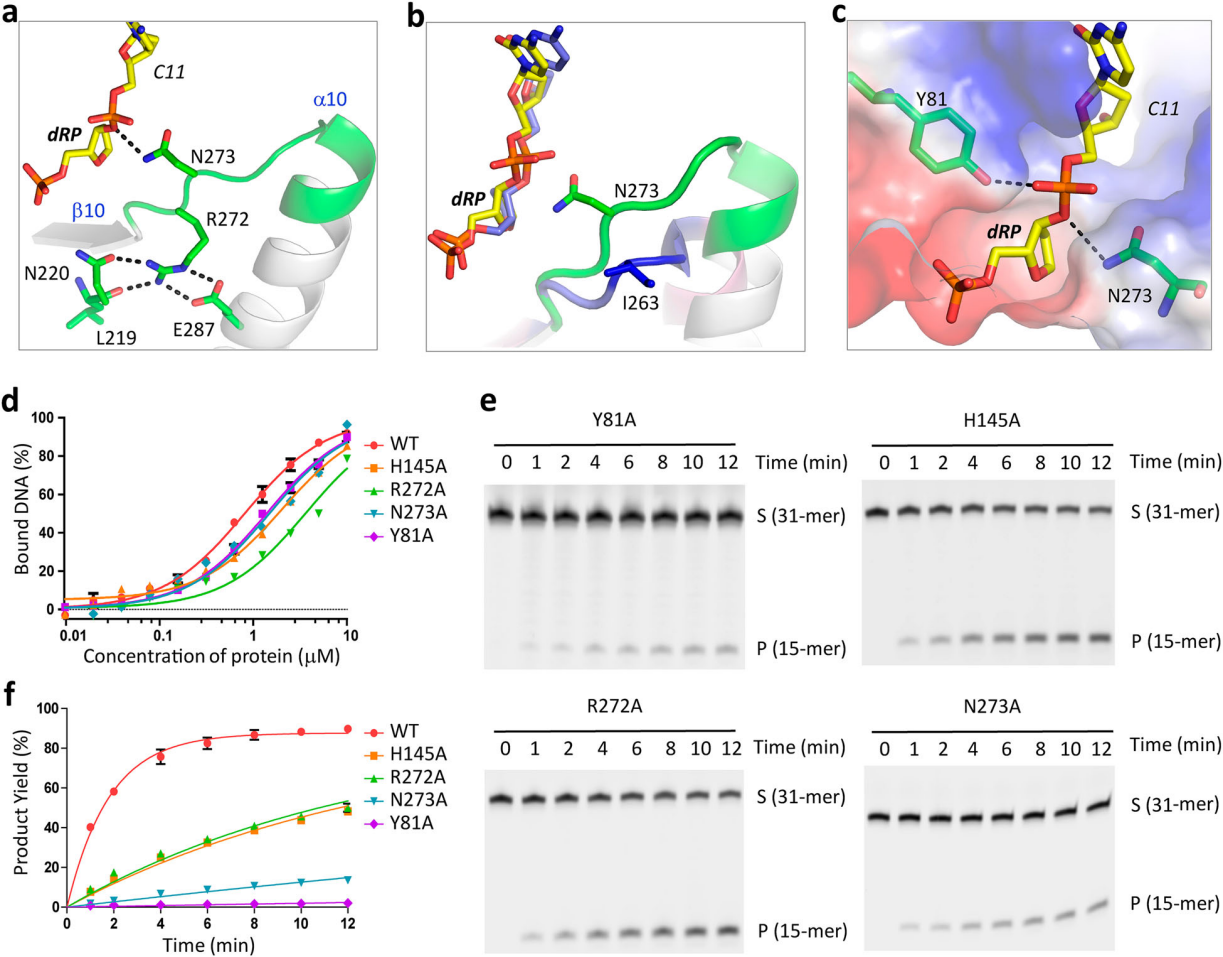
The unique nucleotide-binding pocket of *Asfv*AP. **a** The H-bond interactions maintained by the R3 region residues in the *Asfv*AP/DNA-1 complex. DNA and the R3 region are shown as stick and cartoon, respectively. The C atoms of DNA and the R3 region residues are colored in yellow and green, respectively. **b** Superposition of DNAs and the R3 regions observed in the *Asfv*AP/DNA-1 and *Ec*Nfo/substrate complexes. *Asfv*AP/DNA-1 complex is colored as in **a**, the C atoms are colored in blue for both DNA and the R3 region in the *Ec*Nfo/substrate complex. **c** Surface presentation showing the nucleotide-binding pocket of *Asfv*AP. **d** Comparison of DNA binding by WT *Asfv*AP and mutant proteins. **e, f** SDS-PAGE gel analysis and comparison of DNA-3 cleavage by WT and mutated *Asfv*AP. The substrate and product bands are labeled by S and P, respectively. In panels **d**, **f**, the data represent the mean of three independent experiments. The standard deviation (±SD) values are indicated by error bars. All SDS-Page gel analysis were repeated for at least three times, the representative gels were shown in panels **e**.

The R3 region of *Asfv*AP is 2-amino-acid shorter than that of *Ba*Nfo, but significantly longer than those of *Mt*EndoIV, *Tt*Nfo and *Ec*Nfo (Fig. 1a). Besides Asn273, which forms direct H-bond interaction with dRP in the *Asfv*AP/DNA-1 complex (Fig. 3e), the neighboring Arg272 residue is also unique to *Asfv*AP. The side chain of Arg272 forms four H-bond interactions with the surrounding residues, including one with the main chain of Leu219, one with the side chain of Asn220, and two with the side chain of Glu287 (Fig. 6a). Although Asn220 is highly conserved, Arg272 of *Asfv*AP is replaced by one Thr residue in *Ec*Nfo and other class II AP endonucleases (Fig. 1a). Unlike Arg, the side chain of Thr is too short to form direct H-bond interaction with the conserved Asn residue. Compared to Arg272 in the *Asfv*AP/DNA-1 complex, the backbone of the corresponding Thr262 is shifted 3.0 Å away from the dRP in the *Ec*Nfo/substrate structure. In the homologous protein structures, the R3 region forms one loop (Fig. 4a, b and Supplementary Fig. S3). Asn273 in *Asfv*AP is substituted by residues Pro, Pro, Ala, and Ile in *Ba*Nfo, *Tt*Nfo *Mt*EndoIV and *Ec*Nfo, which are all hydrophobic and incapable of forming H-bond interaction with DNA. As revealed by structural superposition, the backbone of Ile263 of *Ec*Nfo is shifted more than 4.0 Å away from the corresponding Asn273 of *Asfv*AP (Fig. 6b).

The dRP nucleotides adopt similar conformations in *Asfv*AP/DNA-1 and the *Ec*Nfo/substrate complex structures (Figs. 4d and 6b). The sugar pucker of dRP was bound in one shallow pocket, and the bottom of the pocket is formed by the side chains of Phe5, Phe45, and Glu271 in *Asfv*AP. For *Ec*Nfo, the bottom of the dRP-binding pocket is formed by His7, Phe32, and Glu261. The gate to the pocket is formed by the side chains of Tyr81 and Asn273 in *Asfv*AP (Fig. 6c). Tyr81 is highly conserved (Fig. 1a) and adopts similar conformation with the corresponding Tyr72 in the *Ec*Nfo/substrate complex (Supplementary Fig. S5). However, due to the different conformation of the R3 region residues, especially Asn273 in *Asfv*AP and Ile263 in *Ec*Nfo, the nucleotide-binding pocket of *Asfv*AP is much narrower than that of *Ec*Nfo.

To test the functional importance of the gating residues, we constructed two *Asfv*AP mutants (Y81A and N273A) and performed in vitro DNA binding assays (Fig. 6d). Surprisingly, the DNA binding affinities of Y81A mutant (1.39 ± 0.05 μM) and N273A mutant (1.49 ± 0.06 μM) were only slightly weaker than that of WT *Asfv*AP (Supplementary Table S4). Puzzled by these observations, we performed in vitro cleavage assays (Fig. 6e, f) and found that the DNA cleavage activities of Y81A and N273A mutants are about 240-fold and 35-fold weaker than that of WT *Asfv*AP, respectively. These data suggested that the gating residues Tyr81 and Asn273 may play pivotal role in catalytic complex assembly of *Asfv*AP rather than in DNA binding.

As aforementioned, His145 in the R2 loop forms one water-bridged H-bond interaction with the 5′-phosphate group of dRP (Fig. 3e). Although Arg272 of the R3 region does not interact with dRP, it forms extensive H-bond interactions with the surrounding residues, which may help maintain the proper conformation of Asn273. To investigate the function of His145 and Arg272 of *Asfv*AP, we constructed two mutants, H145A and R272A. As showed in Fig. 6e, f, the DNA binding affinities of H145A and R272A mutants were 2.5-fold and 4.5-fold weaker than that of WT *Asfv*AP, respectively. Although the DNA cleavage activities of the two mutants were comparable to each other, both were 7-fold weaker than that of WT *Asfv*AP. Hence, our data suggested that His145 and Arg272 are all important for the function of *Asfv*AP.

### *Asfv*AP has no NIR activity against regular mispairs

In addition to AP endonuclease activity, previous studies reported that *Asfv*AP also possessed NIR activity against certain DNAs, such as DNA containing 5,6-dihydrothymine (DHT) opposite to adenine^24^. However, probably due to the narrower nucleotide-binding pocket (Fig. 6c), the NIR activity of *Asfv*AP was significantly weaker than those of *Ec*Nfo and other class II AP endonucleases. As revealed by our *Asfv*AP/DNA-2 structure, *Asfv*AP has no NIR activity against A:G mispair (Fig. 5d). To verify this observation and to investigate whether *Asfv*AP has NIR activity against other mispairs, we synthesized a set of DNAs (DNA-*XY*: 5′-GGTAAGGGCAGCGTCCXCGACGAGGAATGCA-FAM-3′, 3′-CCATTCCCGTCGCAGGYGCTGCTCCTTACGT-5′, where *X* and *Y* denote either A, G, C, or T). The in vitro DNA cleavage assays showed that *Asfv*AP had no NIR activity against any regular mispairs embedded in the middle of the substrates (Supplementary Fig. S6a). With the exception of the nucleotides at the *X* and *Y* positions, the sequences of DNA-*XY* are identical to those of DNA-3 used in the AP endonuclease assay. Interestingly, although no NIR activity was found for DNA-3 (Fig. 1d), *Asfv*AP exhibited some weak 3′→5′ exonuclease activity against DNA-*XY* substrates (Supplementary Fig. S6a). Recently, Morera and Ishchenko reported one crystal structure of *Ec*Nfo (PDB: 4K1G), which is in complex with dsDNA containing an α-anomeric 2ʹ-deoxyadenosine•T base pair^30^. This structure revealed the detailed basis underlying the exonuclease activity of *Ec*Nfo (Supplementary Fig. S6b–c). We believed that *Asfv*AP may follow the same mechanism in degradation of the terminal nucleotides, since they share the conserved catalytic residues (Figs. 1a and 4d). Rather than substrate-bound or product-bound state, structural superposition suggested that our *Asfv*AP/DNA-2 complex may represent one product-releasing state.

## Discussion

Although ASFV has caused a substantial loss in agricultural industry in the world over the past century, no vaccine or other useful treatment against this virus has been developed so far. Therefore, it will remain a considerable challenge in the foreseeable future. Better understanding of the structures and unique features of functionally important proteins will be helpful to develop the vaccine and treatment against ASFV. *Asfv*AP is one key enzyme involved in the BER pathway of ASFV and is essential for ASFV survival in the host cells. In this study, by solving two complex structures of *Asfv*AP and carrying out binding and cleavage assays, we identified many unique structural features of *Asfv*AP. And, as revealed by structural comparison, we found that *Asfv*AP adopts one novel DNA-binding mode. Besides ASFV, class II AP endonucleases are also expressed by many other viruses, such as Acanthamoeba polyphaga mimivirus, Faustovirus ST1, Harvfovirus sp., Kaumoebavirus, Klosneuvirus KNV1, Moumouvirus australiensis, Moumouvirus goulette, Pacmanvirus A23, Satyrvirus sp, and Terrestrivirus sp. Our studies on *Asfv*AP may also advance our understanding on these viral proteins.

Compared to the homologous proteins, the nucleotide-binding pocket of *Asfv*AP is much narrower and is more specific for the abasic site. Unlike abasic site, other damaged or modified nucleotides, such as DHT, 5,6-dihydrouracil (DHU), and 5-hydroxycytosine (5ohC), are all poor substrates of *Asfv*AP. Normally, the nucleobases of these damaged or modified nucleotides will be removed by DNA glycosylase, forming abasic site. *Asfv*AP cleaves DNA at the 5′ side of the abasic site, generating 3′-OH group and 5′-dRP. As supported by the *Asfv*AP/DNA-1 complex, *Asfv*AP will not remove dRP from the product strand (Fig. 2b, c). *Asfv*AP does not recognize and cleave the mispairs embedded in the middle of the DNA duplex (Supplementary Fig. S6a), but it is able to remove the nucleotides (either paired or mispaired) from the 3′ end or nick site of the DNAs in the absence of abasic sites. The gap is then filled by *Asfv*PolX and sealed by *Asfv*LIG subsequently. As the fidelities of *Asfv*PolX and *Asfv*LIG are very low, they may introduce and tolerate various mispairs at their active sites. Like the AP endonuclease activity, the weak 3′ processing activity of *Asfv*AP may also play a role in the mutagenesis and genotype formation of ASFV.

ASFV is one of the most complex DNA viruses known to date, and encodes more than 150 proteins. As demonstrated by our present and previous studies, *Asfv*AP, *Asfv*PolX and *Asfv*LIG all possess some unique structural features. Besides these DNA repair proteins, the structures of calcineurin inhibitor protein A238L^31^, Bcl-2 like protein A179L^32^ and sulfhydryl oxidase B119L^33^ of ASFV have also been reported previously. However, the structures of the majority of ASFV proteins are currently unavailable. In the future, it is worth to investigate whether certain ASFV proteins possess unusual features and are capable of cleaning dRP and damaged nucleobases.

Recently, some poly ADP-ribose polymerase (PARP) inhibitors, which can selectively target the DNA repair defect in hereditary breast cancer, have been discovered^34^. The success of the PARP inhibitors has stimulated the development of inhibitors targeting other repair enzymes, such as DNA ligases^35–37^. As reported previously, deletion of *Asfv*AP coding gene significantly lowered the replication of ASFV, implying that the BER pathway plays a critical role in the life cycle of ASFV. Similar to *Asfv*PolX and *Asfv*LIG, *Asfv*AP also possesses many unique structural features, especially the narrower nucleotide-binding pocket at the active site, and these unique features may serve as an ideal target for designing small molecule inhibitors. Unlike *Asfv*AP, the AP endonuclease of the pig (*Sus*APE) belongs to calss I AP endonuclease family, and is closely related (>90% nucleotide similarity) with human APE1 (*Hs*APE1). As revealed by *Hs*APE1^38^ and many other homologous protein structures, the overall fold of class I AP endonucleases is very different from that of *Asfv*AP. Hence, the *Asfv*AP-specific inhibitors will not interfere with the normal function of *Sus*APE, but they will impair the ASFV repair process and help combat this deadly virus in the future.

## Materials and methods

### Plasmid construction

The genes containing the codon-optimized cDNA sequences of WT *Asfv*AP (Supplementary Table S5) and *Ec*Nfo (Supplementary Table S6) were purchased from Shanghai Geneways Biotech Co., Ltd, China. The target fragment was recovered and recombined into pET28-Sumo vector. The recombinant vectors (coding for His-Sumo-*Asfv*AP or His-Sumo-*Ec*Nfo) were then transformed into *Escherichia coli* BL21 DE3 competent cells for protein expression. The recombinant His-Sumo-*Asfv*AP coding vector was utilized as template during the plasmid constructions of all *Asfv*AP mutants, via overlap polymerase chain reactions or site direct mutagenesis according to the manufacturer’s protocols. The detailed sequences of the primers are listed in Supplementary Table S5.

### Protein expression and purification

WT and all mutant *Asfv*AP proteins were expressed and purified using the same procedures. Briefly, the frozen recombinant strains were revived in Lysogeny broth (LB) medium supplemented with 50 μg/mL kanamycin at 37 °C overnight. Every 20 mL revived bacterium suspension was inoculated into 1 L LB medium supplemented with kanamycin (50 μg/mL) and ZnCl_2_ (0.2 mM), cultured at 37 °C with continuous shaking. Protein expression was induced at OD_600_ ≈ 0.6 by adding of isopropyl β-D-1-thiogalacto-pyranoside (IPTG) at a final concentration of 0.05 mM. The induced cultures were then grown at 18 °C for an additional 18 h.

The cells were harvested by centrifugation, the pellets were resuspended in Buffer A (20 mM MES pH 6.0, 500 mM NaCl, 25 mM imidazole) and lysed under high pressure via a cell crusher. The homogenate was clarified by centrifugation and the supernatant was loaded onto a HisTrap^TM^ HP column equilibrated with Buffer A. The fusion protein was eluted from the column using Buffer B (20 mM MES pH 6.0, 500 mM NaCl, 500 mM imidazole) with a gradient. The fractions containing the desired fusion proteins were pooled and dialyzed against Buffer C (20 mM MES pH 6.0, 500 mM NaCl) at 4 °C for 1 h; Ulp1 protease was added to the sample during the dialysis process. The sample was applied to a HiTrap SP HP column equilibrated with Buffer D (20 mM MES pH 6.0, 100 mM NaCl) and eluted using Buffer E (20 mM MES pH 6.0, 1 M NaCl) with a continuous gradient. The target protein was concentrated and loaded onto a Hi 16/60 Superdex G200 column equilibrated with gel filtration buffer (20 mM MES pH 6.0, 200 mM NaCl).

*Ec*Nfo was expressed using similar procedures as *Asfv*AP. The cell pellets were harvest and resuspended in Buffer F (20 mM Tris pH 8.0, 500 mM NaCl, 25 mM imidazole) and lysed under high pressure. The homogenate was clarified by centrifugation and the supernatant was loaded onto a HisTrap^TM^ HP column equilibrated with Buffer F. The fusion protein was eluted from the column using Buffer G (20 mM Tris pH 8.0, 500 mM NaCl, 500 mM imidazole) with a gradient. The fractions containing the desired proteins were pooled and dialyzed against Buffer F at 4 °C for 1 h; Ulp1 protease was also added to the sample during the dialysis process. The sample was applied to a HiTrap QP HP column equilibrated with Buffer H (20 mM Tris pH 8.0, 100 mM NaCl) and eluted using Buffer E with a continuous gradient.

### In vitro DNA binding and cleavage assays

All DNAs used in the binding and cleavage assays were purchased from Shanghai Generay Biotech Co., Ltd. The DNA substrates were assembled in a molar ratio of 1:1 in reaction buffer (20 mM MES pH 5.0–7.0 or 20 mM Tris pH 7.5–8.0, 200 mM NaCl, 5 mM MgCl_2_, 0.05% NP40, 5% Glycerol). For cleavage assays, a 50-μL reaction system (composed of 40 μL gel filtration buffer, 5 μL 2 μM DNA, and 5 μL protein) was established. The final protein concentrations are 0.025 μM. The reactions (5 μL) were carried out at 37 °C and quenched by adding of 5 μL termination buffer (90% formamide, 20 mM EDTA, 0.05% bromophenol blue, and 0.05% xylene blue) at various time points. Eight μL samples were loaded onto pre-warmed 18% urea sequencing gels and run at 18–20 W and 40–45 °C for 60 min. The gel was visualized using Typhoon FLA 9000, and intensities of the substrate and product bonds were quantified by ImageQuantTL. Data were then fitted to the exponential $Y=Y_{\max}[1-e^{(-K_{\rm{obs}}t)}]$ using non-linear regression in GraphPad Prism 5. The observed rate constant (*K*_obs_) and maximum cleavage yield (*Y*_max_) were determined from the regression curve (Supplementary Tables S2–4).

Substrates used in fluorescence polarization were diluted to 20 nM with reaction buffer. A 200-μL reaction system, composed of 100 μL DNA (20 nM) and 100 μL proteins was established. The final protein concentration was fixed at 10 nM. The samples were incubated at 0 °C for 30 min. All FP measurements were performed at room temperature in the dark using Synergy 2 Muti-Mode Microplate Reader (BioTek). The data were fitted to the exponential *Y* = Bottom + (Top−Bottom)/(1 + 10^(LogEC50-*X*)^) using non-linear regression in GraphPad Prism. The *K*_d_ values (Supplementary Table S4) were determined from the regression curve. All the experiments were repeat for at least three times.

### Crystallization and data collection

All DNAs used in the structural studies were dissolved in ddH_2_O. The crystallization samples were prepared by mixing *Asfv*AP and DNA-1 or DNA-2 together at room temperature. The final concentration of the protein is 0.2 mM, and the concentration of DNA-1 or DNA-2 duplexes is 0.22 mM for the crystallization attempt. The initial crystallization conditions were identified at 18 °C using the crystallization robot system and commercial crystallization kits. During initial screening, the sitting–drop vapor diffusion method was used, whereas all the crystal optimization procedures were performed using the hanging-drop vapor diffusion method. The *Asfv*AP/DNA-1 crystals were grown under the condition composed of 0.1 M BIS-TRIS pH 5.5, 13% w/v PEG 10,000 and 0.1 M ammonium acetate. The crystallization condition of AsfvAP/DNA-2 is composed of 16% w/v PEG 3350 and 0.1 M potassium sodium tartrate tetrahydrate.

Both *Asfv*AP/DNA-1 and *Asfv*AP/DNA-2 crystals were cryoprotected using their mother liquor supplemented with 25% glycerol and snap-frozen in liquid nitrogen. X-ray diffraction data were collected on beamline BL17U1, BL18U1, and BL19U1 at the Shanghai Synchrotron Radiation Facility (SSRF). Data processing was carried out using the HKL2000 or HKL3000 programs^39^. The data collection and processing statistics are summarized in Supplementary Table S1.

### Structure determination and refinement

The *AsfvAP*/DNA-1 structure was solved by single-wavelength anomalous diffraction (SAD) method^40^ using the anomalous signal of Zn^2+^ cofactor ions, which were co-purified with *Asfv*AP protein. The AutoSol program^41^ embedded in the Phenix suite^42^ was utilized to determine the structure, which identified all the six Zn^2+^ ions within the asymmetric unit. The phasing figure-of-merit (FOM) value is 0.35. The initial model, which covers approximately 60% of protein residues in the asymmetric unit, was built using the Autobuild program and was refined against the diffraction data using the Refmac5 program^43^ of CCP4i^44^. During refinement, 5% of randomly selected data was set aside for free R-factor cross validation calculations. The 2F_o_–F_c_ and F_o_–F_c_ electron density maps were regularly calculated and used as guides for building the missing amino acids, DNA, and solvent molecules using COOT^45^. The *AsfvAP*/DNA-2 structure was solved using the molecular replacement (MR) method with the Phaser program of the CCP4i suite; the *Asfv*AP molecule of the *AsfvAP*/DNA-1 structure was used as the search model. The final refinement of all structures was done using the phenix.refine program^46^ of Phenix. The structural refinement statistics are summarized in Supplementary Table S1.

### Accession codes

Structural factors and coordinates have been deposited in the Protein Data Bank under accession codes 6KI3 and 6KHY for the *Asfv*AP/DNA-1 and *Asfv*AP/DNA-2 complex structures, respectively.

## Supplementary information

Supplementary information, Figures and Tables

## Supplementary information

**Supplementary Information** accompanies the paper at (10.1038/s41421-020-0146-2).
