# Supplementary material for: A unique DNA-binding mode of African swine fever virus AP endonuclease
Source: Cell Discov. 2020 Mar 17;6:13. doi: 10.1038/s41421-020-0146-2 (PMC7076025; doi:10.1038/s41421-020-0146-2)
Supplement: Supplementary file 1 — Supplementary information, Figures and Tables [file 41421_2020_146_MOESM1_ESM.pdf]

Supplementary Information
for

A unique DNA-binding mode of African swine fever virus AP endonuclease

Yiqing Chen[1,#], Xi Chen[1,#], Qi Huang[1], Zhiwei Shao[1], Yanqing Gao[1], Yangyang Li[1], Chun Yang[1], Hehua Liu[1], Jixi Li[1], Qiyao Wang[2], Jinbiao Ma[1], Yong-Zhen Zhang[3,4,*], Yijun Gu[5,*], Jianhua Gan[1,*]

[1]State Key Laboratory of Genetic Engineering, Collaborative Innovation Center of Genetics and Development, Shanghai Public Health Clinical Center, School of Life Sciences, Fudan University, Shanghai 200438, China

[2]State Key Laboratory of Bioreactor Engineering, East China University of Science and Technology, Shanghai 200237, China

[3]Shanghai Public Health Clinical Center, School of Life Sciences, Fudan University, Shanghai 200438, China

[4]State Key Laboratory for Infectious Disease Prevention and Control, Collaborative Innovation Center for Diagnosis and Treatment of Infectious Diseases, National Institute for Communicable Disease Control and Prevention, Chinese Center for Disease Control and Prevention, Changping, Beijing 102206, China

[5]National Center for Protein Science Shanghai, Shanghai Advanced Research Institute, Chinese Academy of Sciences, Shanghai 201210, China

***Correspondence to**: Yong-Zhen Zhang (zhangyongzhen@icdc.cn) or Yijun Gu (guyijun@sari.ac.cn) or Jianhua Gan (ganjhh@fudan.edu.cn)

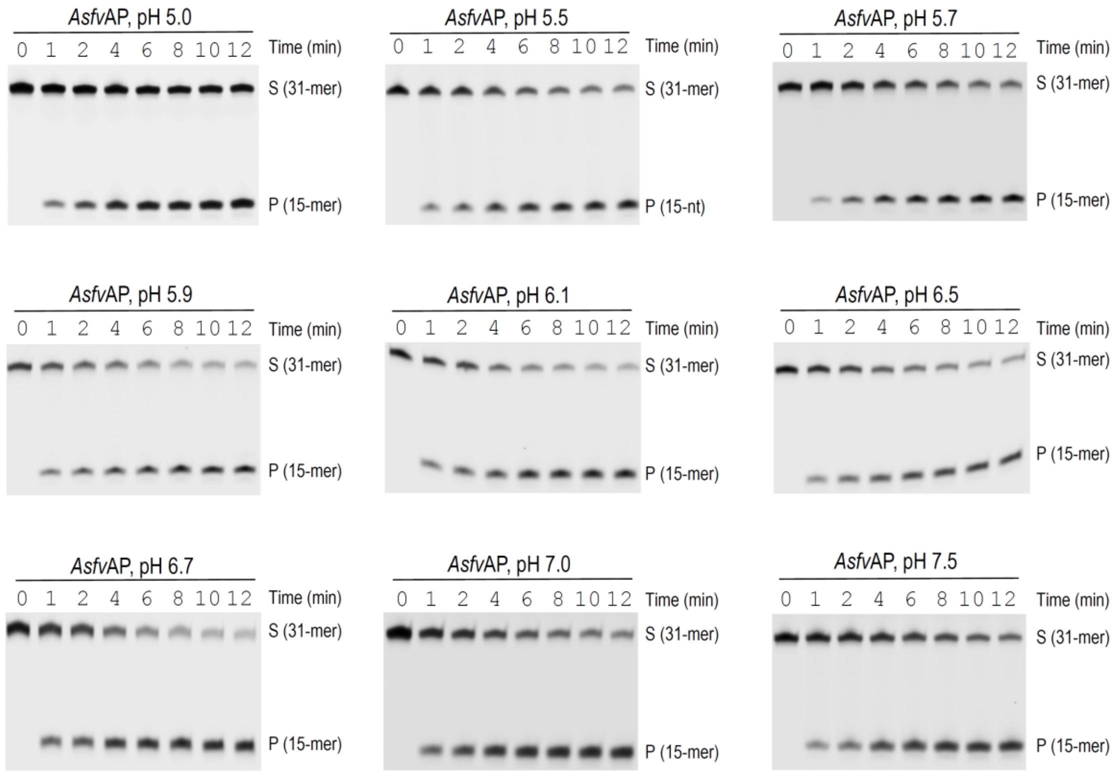

Supplementary Fig. S1: SDS-PAGE gel analysis showing the impacts of pH value on the catalytic efficiency of *Asfv*AP. FAM-labelled DNA-3 was used in all AP endonuclease assays. The substrate and product bands are labelled by S and P, respectively. All SDS-Page gel analysis were repeated for at least three times, the representative gels were shown in the figure.

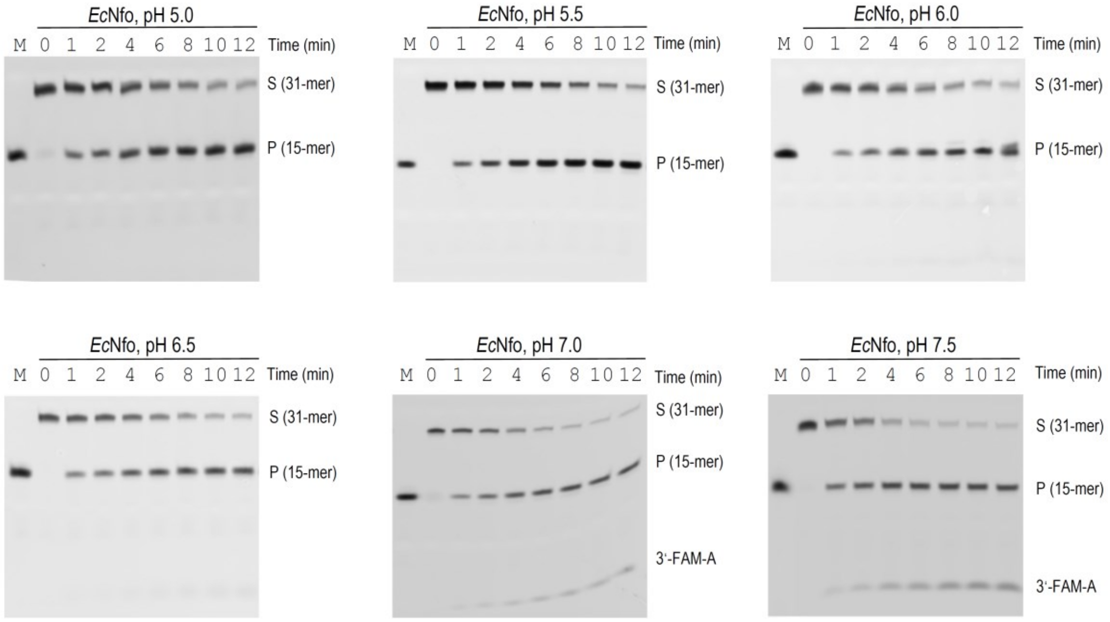

Supplementary Fig. S2: SDS-PAGE gel analysis showing the impacts of pH value on the catalytic efficiency of *Ec*Nfo. FAM-labelled DNA-3 was used in all AP endonuclease assays. The substrate and product bands are labelled by S and P, respectively. All SDS-Page gel analysis were repeated for at least three times, the representative gels were shown in the figure.

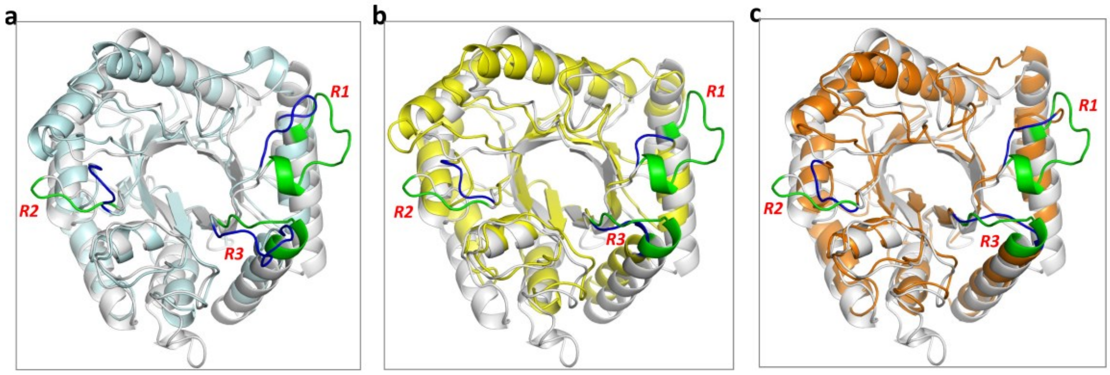

Supplementary Fig. S3: Structural comparison between *Asfv*AP and homologous proteins. (**a-c**) Superposition of *Asfv*AP with *Ba*Nfo, *Mt*EndoIV, and *Tt*Nfo, respectively. *Asfv*AP are colored in white in all panels. *Ba*Nfo, *Mt*EndoIV and *Tt*Nfo are colored in cyan, yellow, and orange, respectively. The R1-R3 regions are colored in green for *Asfv*AP, whereas they are colored in blue for all other proteins.

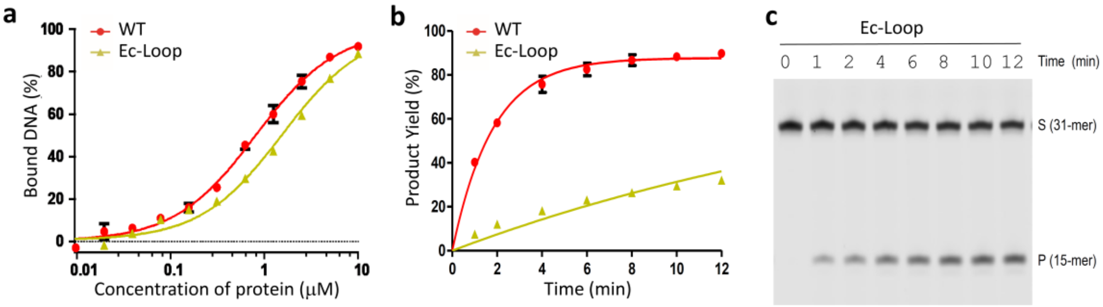

Supplementary Fig. S4: Comparison of (**a**) *in vitro* DNA binding and (**b**) DNA cleavage by WT and Ec-Loop mutant of *Asfv*AP. (**c**) SDS-PAGE gel analysis showing DNA cleavage by Ec-Loop mutant of *Asfv*AP. The substrate and product bands are labelled by S and P, respectively. In panels (**a**) and (**b**), the data represent the mean of three independent experiments. The standard deviation (±SD) values are indicated by error bars. All SDS-Page gel analysis were repeated for at least three times, the representative gels were shown in panels (**c**).

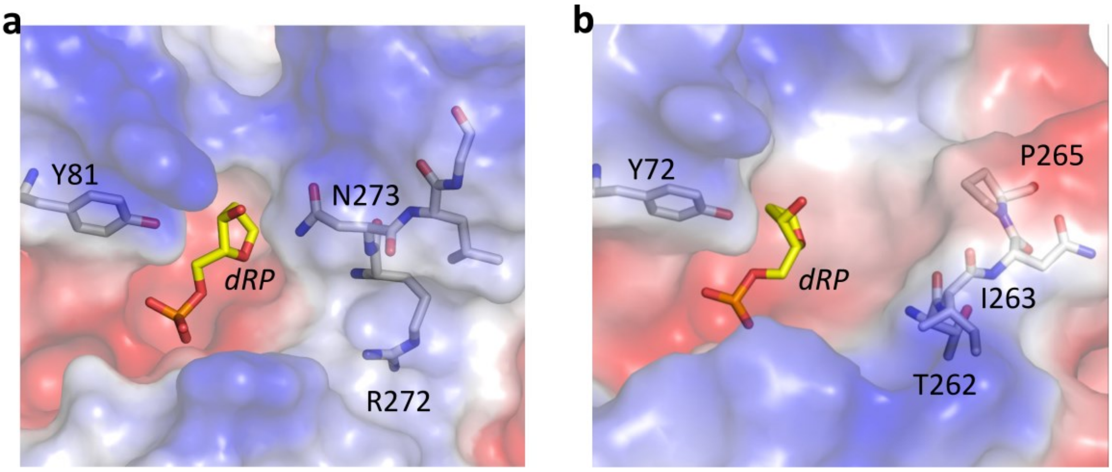

Supplementary Fig. S5: Comparison of the nucleotide-binding pockets observed in (**a**) the *Asfv*AP/DNA-1 complex and (**b**) the *Ec*Nfo/substrate complex.

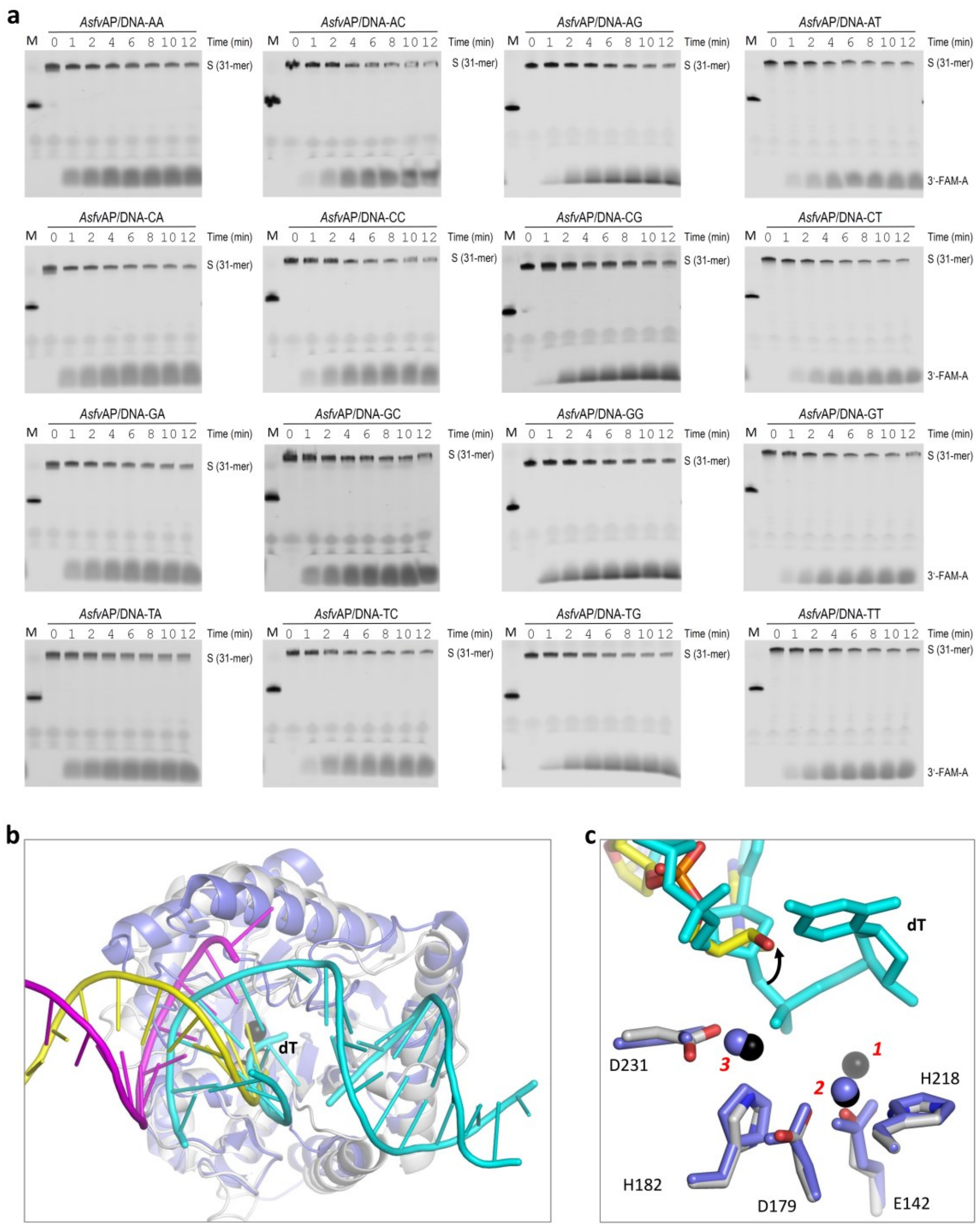

Supplementary Fig. S6: (**a**) SDS-PAGE gel analysis showing DNA cleavage by *Asfv*AP. FAM-labelled DNA-XY (5'-GGTAAGGGCAGCGTCCXCGACGAGGAATGCA-FAM-3', 3'-CCATTCCCGTCGCAGGYGCTGCTCCTTACGT-5') was used in the cleavage assays. DNA marker (5'-FCGACGAGGAATGCA-FAM-3') is labelled as M. All SDS-Page gel analysis were repeated for at least three times, the representative gels were shown in the figure. (**b**-**c**) Superposition of *Asfv*AP/DNA-2 and *Ec*Nfo/dsDNA (PDB:

4K1G) complexes. In panel (**b**), *Asfv*AP and *Ec*Nfo are colored in white and light-blue, respectively. For the *Asfv*AP/DNA-2 complex, the two DNA strands are colored in yellow and magenta, respectively. For the *Ec*Nfo/dsDNA complex, DNA strands are colored in cyan. In panel (**c**), the C-atoms of *Asfv*AP and DNA are colored in white and yellow in the *Asfv*AP/DNA-2 complex. The *Ec*Nfo/dsDNA complex is colored as in panel (**b**). The Zn^{2+} ions are colored in black and light-blue in the *Asfv*AP/DNA-2 and *Ec*Nfo/dsDNA complexes, respectively.

Supplementary Table S1. Data collection and refinement statistics

Structure<br>(PDB ID)	*Asfv*AP/DNA-1<br>6KI3	*Asfv*AP/DNA-2<br>6KHY
**Data collection**[a]		
Space group	$P2_1$	$C222_1$
Cell parameter		
a, b, c (Å)	50.6, 84.7, 98.6	110.5, 148.5, 178.1
α, β, γ (°)	90.0, 93.7, 90.0	90.0, 90.0, 90.0
Wavelength (Å)	0.9793	0.9793
Resolution (Å)	30.0-2.35	30.0-3.0
Last shell (Å)	2.39-2.35	3.11-3.0
Completeness (%)	98.6(95.9)	95.1(88.0)
Redundancy	3.3(2.6)	4.0(2.5）
I/σ(I)	8.5(2.1)	12.0(1.8)
CC1/2	0.963(0.523)	0.991(0.573)
R_{merge} (%)	13.7(52.6)	8.5(36.2)
**Refinement**		
Resolution (Å)	28.3-2.35	29.9-3.0
R_{work} (%) / R_{free} (%)	20.7/24.4	21.1/24.9
No. of atoms		
Protein	4575	9209
DNA	1358	2002
Water	88	20
Zinc ion	6	12
Wilson B factors ($Å^2$)	38.8	59.5
R.m.s. deviations		
Bond length (Å)	0.003	0.003
Bond angle (°)	0.570	0.554
Ramachandran plot (%)		
Most favored	96.6	95.6
Additional allowed	3.4	4.4
Molprobity clash score	6.64	5.83

Supplementary Table S2. Effects of pH values on the catalytic efficiency of *Asfv*AP

enzyme	pH value	K_{obs} (×10^{-3}, min^{-1}）[a]
*Asfv*AP	5.0	0.077±0.001
*Asfv*AP	5.5	0.137±0.011
*Asfv*AP	5.7	0.139±0.005
*Asfv*AP	5.9	0.205±0.013
*Asfv*AP	6.1	0.249±0.014
*Asfv*AP	6.3	0.552±0.024
*Asfv*AP	6.5	0.214±0.011
*Asfv*AP	6.7	0.189±0.007
*Asfv*AP	7.0	0.199±0.016
*Asfv*AP	7.5	0.120±0.012

[a]: Values are means ± s.d. from three independent experiments.

Supplementary Table S3. Effects of pH values on the catalytic efficiency of *Ec*Nfo

enzyme	pH value	K_{obs} (×10^{-3}, min^{-1}) [a]
*Ec*Nfo	5.0	0.204±0.005
*Ec*Nfo	5.5	0.186±0.004
*Ec*Nfo	6.0	0.204±0.004
*Ec*Nfo	6.5	0.238±0.008
*Ec*Nfo	7.0	0.354±0.005
*Ec*Nfo	7.5	0.489±0.012
*Ec*Nfo	8.0	0.652±0.036

[a]: Values are means ± s.d. from three independent experiments.

Supplementary Table S4. Summarization of the DNA binding and catalytic efficiency of WT and mutated *Asfv*AP proteins

enzyme	K_d (M)	K_{obs} ($\times 10^{-3}$, min^{-1}) [a]
*Asfv*AP_WT	0.81±0.14	0.5524±0.0236
*Asfv*AP_H8A	4.83±0.52	0.2718±0.0169
*Asfv*AP_S14A	6.46±0.77	0.2270±0.0101
*Asfv*AP_H8A/S14A	10.25±3.40	0.2429±0.0146
*Asfv*AP_C16A/C20A	5.27±0.55	0.2863±0.0185
*Asfv*AP_Ec-Loop	1.52±0.07	0.0442±0.0018
*Asfv*AP_Y81A	1.39±0.05	0.0023±0.0001
*Asfv*AP_H145A	2.04±0.06	0.0726±0.0016
*Asfv*AP_H148A/H149A	12.43±0.23	0.1591±0.0150
*Asfv*AP_R272A	3.65±0.06	0.0784±0.0026
*Asfv*AP_N273A	1.49±0.06	0.0155±0.0006

[a]: Values are means ± s.d. from three independent experiments.

Supplementary Table S5. Sequences of the optimized cDNA of wild type *Asfv*AP and the primers for *Asfv*AP mutant constructions

The optimized cDNA sequence of wild type *Asfv*AP [a] (from 5' to 3')

*GGATCC*GGTGGTGGCATGTTCGGTGCGTTTGTTTCTCACCGTCTGTGGTCTGACTCTGGTTGCACCACCACTTG
CATCACCAACTCTATCGCGAACTACGTTGCTTTTGGTGAACAGATCGGTTTCCCGTTCAAATCCGCCCAGGTTT
TCATCGCGGGTCCGCGTAAAGCGGTTATCAACATCCAGGAAGACGACAAAGTTGAACTCCTGAAAATGATTGTT
AAACACAACCTGTGGGTTGTTGCGCACGGTACCTATCTGGACGTTCCGTGGTCTCGTCGCTCTGCTTTCGTTAC
CCATTTCATCCAGCAAGAGCTCCTCATTTGCAAGGAAGTTGGTATCAAAGGTCTGGTTCTGCACCTGGGTGCGG
TTGAACCGGAACTGATCGTAGAAGGTCTCAAAAAGATTAAACCGGTGGAAGGTGTTGTTATCTACCTGGAAACC
CCGCACAACAAACACCACACCTACAAATACTCTACCATGGAACAAATCAAGGAGCTGTTCCTCCGTATCCGTAA
CACCCGTCTGAAGCAGATTGGTCTGTGCATCGACACGGCGCACATCTGGTCTTCTGGTGTAAACATCTCTTCTT
ACAACGACGCCGGTCAGTGGCTGCGTTCCCTGGAGAATATCCACTCTGTTATTCCGCCGTCTCACATCATGTTC
CACCTGAACGATGCGGCGACCGAATGCGGTTCTGGTATCGACCGTCACGCGTCTCTGTTCGAAGGTATGATCTG
GAAATCTTACTCTCACAAGATCAAACAGTCTGGCCTGTATTGCTTCGTTGAATACATCACTCGTCACCAGTGCC
CTGCGATCCTGGAACGTAACCTCGGTTCTTCTATGCAACTGCAGACTGCGCTGACCGCGGAATTCACCACCCTG
AAATCTCTGCTCAAGTAA*CTCGAG*

Primers used for *Asfv*AP mutant constructions

Name	Sequence (from 5' to 3')
AP_F	CGCGGATCCGGTGGTGGCATGTTCGGTGCGTTT
AP_R	CCGCTCGAGTTACTTGAGCAGAGATTTCA
C16A/C20A_F	CGCGGATCCGGTGGTGGCATGTTCGGTGCGTTTGTTTCTCACCGTCTGTGGTC TGACTCTGGTGCCACCACCACTGCCATCACCAA
Ec-loop_F	GGATCCGGTGGTGGCATGTTCGGTGCGTTTGTTTCTGCTGCGGGTGGCATCAC CAACTCTATCGCGAACTACGTTGCTTTTGGT
H8A_F	GGTGCGTTTGTTTCTGCCCGTCTGTGGTCTGAC
H8A_R	GTCAGACCACAGACGGGCAGAAACAAACGCACC
S14A_F	CGTCTGTGGTCTGACGCCGGTTGCACCACCACT
S14A_R	AGTGGTGGTGCAACCGGCGTCAGACCACAGACG
Y81A_F	GTTGCGCACGGTACCGCCCTGGACGTTCCGTGG
Y81A_R	CCACGGAACGTCCAGGGCGGTACCGTGCGCAAC
H145A_F	TACCTGGAAACCCCGGCCAACAAACACCACACC
H145A_R	GGTGTGGTGTTTGTTGGCCGGGGTTTCCAGGTA
H148A/H149A_F	ACCCCGCACAACAAAGCCGCCACCTACAAATACTCT
H148A/H149A_R	AGAGTATTTGTAGGTGGCGGCTTTGTTGTGCGGGGT
R272A_F	CCTGCGATCCTGGAAGCCAACCTCGGTTCTTCT
R272A_R	AGAAGAACCGAGGTTGGCTTCCAGGATCGCAGG
N273A_F	GCGATCCTGGAACGTGCCCTCGGTTCTTCTATG
N273A_R	CATAGAAGAACCGAGGGCACGTTCCAGGATCGC

[a]: GGATCC and CTCGAG at the 5'-end and 3'-end are BamHI and XhoI recognition sequence.

Supplementary Table S6. Sequences of the optimized cDNA of *Ec*Nfo

**The optimized cDNA sequence of *Ec*Nfo [a]** (from 5' to 3')
*GGATCC*ATGAAGTATATCGGTGCTCACGTTTCTGCCGCTGGTGGTCTCGCTAACGCAGCGATCCGTGCGGCCGA<br>AATCGACGCGACCGCGTTCGCGCTCTTCACCAAAAACCAGCGTCAGTGGCGTGCAGCCCCACTCACCACCCAGA<br>CTATCGACGAATTCAAGGCAGCCTGCGAAAAATACCACTACACCTCCGCGCAGATCCTGCCGCATGACTCTTAC<br>CTCATCAACCTCGGTCATCCGGTCACCGAAGCACTCGAAAAGTCTCGTGACGCGTTCATTGACGAAATGCAGCG<br>TTGCGAACAGCTGGGTCTGTCTCTGCTGAATTTCCACCCTGGCTCTCACCTGATGCAGATCTCTGAAGAAGACT<br>GCCTGGCGCGCATCGCGGAAAGCATCAACATCGCGCTGGACAAAACCCAAGGTGTTACCGCTGTGATCGAAAAC<br>ACCGCGGGTCAGGGTTCTAACCTGGGCTTCAAATTCGAACACCTGGCGGCGATTATCGATGGTGTGGAAGACAA<br>ATCTCGCGTTGGTGTCTGTATCGACACCTGCCATGCATTTGCTGCTGGCTACGACCTGCGCACGCCTGCGGAAT<br>GCGAGAAGACCTTCGCGGACTTCGCGCGTACCGTTGGTTTCAAATACCTGCGTGGTATGCACCTGAACGACGCA<br>AAATCTACCTTTGGTTCCCGTGTTGACCGTCACCACTCTCTCGGTGAAGGTAACATTGGCCACGATGCGTTCCG<br>TTGGATCATGCAGGACGACCGTTTCGACGGTATCCCGCTGATCCTGGAAACCATTAACCCGGACATCTGGGCGG<br>AAGAAATCGCGTGGCTCAAAGCGCAACAGACCGAAAAGGCGGTTGCGTAA*CTCGAG*

[a]: GGATCC and CTCGAG at the 5'-end and 3'-end are BamHI and XhoI recognition sequence.
